# Efficacy of mHealth aided 12-week meditation and breath intervention on change in burnout and professional quality of life among health care providers of a tertiary care hospital in north India: a randomized waitlist-controlled trial

**DOI:** 10.3389/fpubh.2023.1258330

**Published:** 2023-11-01

**Authors:** Praag Bhardwaj, Monika Pathania, Yogesh Bahurupi, Divya Kanchibhotla, Prateek Harsora, Vyas Kumar Rathaur

**Affiliations:** ^1^Deparment of Medicine, All India Institute of Medical Sciences, Rishikesh, India; ^2^Deparment of Community and Family Medicine, All India Institute of Medical Sciences, Rishikesh, India; ^3^Sri Sri Institute of Advanced Research, Bengaluru, India; ^4^Veer Chandra Singh Garhwali Government Institute of Medical Science and Research, Srinagar, Uttarakhand, India

**Keywords:** mental health, mind body medicine, occupational health, physician health, pranayama, resilience, stress, tele-medicine

## Abstract

**Introduction:**

Burnout is “Chronic workplace stress that has not been successfully managed.” Professional quality of life (PQL) includes work related experiences of compassion satisfaction and compassion fatigue. Healthcare providers (HCPs) are highly susceptible to burnout and compassion fatigue due to their demanding work, which lowers PQL. Burnout leads to poor care, medical errors, and patient safety across healthcare disciplines. Yoga has been shown to improve resilience, reduce stress, and increase self-compassion and psycho-physiological coherence. This study compared HCPs in a mHealth-aided 12-week yoga-based meditation and breath intervention to waitlist controls for HCP burnout and PQL at a north Indian tertiary care hospital.

**Methods:**

This was randomized waitlist-controlled trial. Total 98 HCPs (62 males and 36 females) with an average age of 28.26 ± 3.547 years were enrolled consecutively from March 2021 to November 2022. Randomization was done with opaque sealed envelopes numbered in a computer-generated sequence. The experimental group (*n* = 49) received 12 online weekly yoga sessions and performed daily home practice (6 days a week). The waitlisted control group (*n* = 49) continued their daily routine. Maslach’s burnout inventory (MBI), professional quality of life (PQL) and anthropometric measurements were assessed at baseline and after 12 weeks.

**Results:**

After 12 weeks, the MBI outcomes of emotional exhaustion, depersonalization, and personal accomplishment showed a highly significant difference between the two groups (*p* < 0.001). PQL outcomes of compassion satisfaction, burnout, and secondary trauma also differed significantly (*p* < 0.001). Within group analysis showed that MBI and PQL outcomes improved significantly (*p* < 0.001) for the experimental group after 12 weeks.

**Conclusion:**

The current study contributes to the existing evidence on the effectiveness of Yoga in managing stress and developing resilience among doctors, nurses, and other medical professionals. Integrating yoga into healthcare settings is crucial for addressing the detrimental impact of burnout on decision-making and promoting positive patient outcomes. mHealth technologies have the potential to enhance the user-friendliness of yoga-based interventions by personalizing the practice space and time. Yoga-based interventions and mHealth technologies can effectively address physician burnout, in a simple and implementable manner.

## 1. Introduction

Burnout occurs when employees are exposed to a stressful work environment with high job demands and low resources (1). As per the World Health Organization and the International Classification of Disease-11, “burnout is an occupational syndrome conceptualized as resulting from chronic workplace stress that has not been successfully managed” (2, 3). Health care providers (HCPs) are particularly susceptible to burnout due to the demanding nature of their job (1).

Burnout manifests in three main dimensions: emotional exhaustion (EE), depersonalization (DP), and low personal accomplishment (PA) (2, 3). EE is characterized by feeling overwhelmed, stressed, and fatigued when the demands of the job outweigh one’s capacity to cope. DP involves losing enthusiasm and displaying an impersonal attitude toward work, perceiving the job as burdensome or monotonous. Low PA refers to experiencing low levels of competence, effectiveness, and impact on work and people.

Burnout is a global phenomenon, with studies predicting a wide range of prevalence estimates among HCPs in Arab countries (4), the Middle East (5), and the US (6). The prevalence of burnout among intensive care professionals ranges from 6 to 47% (7). In India, a questionnaire-based survey showed a high prevalence of burnout among doctors, with scores of 45, 65.98, and 87% for the three Maslach burnout inventory (MBI) subscales of EE, DP, and PA, respectively (8). Another study suggested that residential doctors not only have moderate (66%) to high (13%) levels of stress, but also experience burnout (90%), go through depression (30%), and even have suicidal thoughts (16%). During COVID-19, there was a global surge in the number of cases of burnout, with prevalence counts going as high as 67% (9). This trend was also observed in India, where burnout was rampant among frontline nurses and HCPs (10, 11).

Professional quality of life (PQL) refers to the overall well-being and satisfaction of HCPs with their work. PQL comprises two fundamental dimensions: compassion satisfaction (CS) and compassion fatigue (12). CS is the positive aspect of PQL and refers to the joy of helping others professionally. It also involves feeling good about co-workers, contributing to the workplace, and enjoying one’s work’s larger social impact (12). Compassion fatigue is the negative aspect of PQL, which can be further divided into two components (12). The first component is burnout (BO), which is typically associated with feelings of exhaustion, frustration, anger, and depression. (Hereby BO is referred to as the individual component of Burnout under compassion fatigue of PQL) The second component is known as secondary traumatic stress (ST), which is driven by fear and the experience of work-related trauma (12).

Prevalence trends for PQL among HCPs in India have not been directly examined, and further research is needed in this regard. Nonetheless, the importance of PQL in healthcare settings and understanding its impact on HCPs cannot be undermined.

Burnout and PQL are interlinked. Evidence suggests that self-compassion and resilience are associated with higher PQL, while compassion fatigue and burnout are negatively associated with PQL (13). Burnout can negatively affect the health and well-being of HCPs (13), leading to poor quality care, medical errors, and patient safety issues (7). Higher levels of burnout have been associated with lower quality and safety across healthcare disciplines (14).

The cause of this profound burnout syndrome and the interlinked low PQL among doctors and HCPs can be pin-pointed to prolonged stress due to overwork. However, the factors responsible for this stress accumulation may vary and include a lack of resources, a high patient load, administrative and managerial pressure for profit earning, and crippling dependency upon the hospital, institution, or organization (15, 16). Despite undergoing significant amounts of stress, HCPs in India avoid seeking help due to reasons like lack of time, cost issues, fear of future academic jeopardy, confidentiality issues, and stigma (17).

Mind–body therapies like Yoga and mindfulness, among others, have been recognized as helpful interventions to mitigate the effects of stress and reduce burnout among HCPs (18). Yoga is a system of Indian philosophy that emphasizes the balance of physical, mental, and spiritual health (19). Nowadays, Yoga is commonly known as a multimodal mind–body practice that includes different techniques such as physical postures, breathing practices, philosophical wisdom, and meditation (20). Several physical and mental health benefits have been associated with Yoga, including improvements in physical performance and injury prevention (21), adopting a healthier lifestyle (22), physical and mental well-being (20), etc.

Yoga is becoming increasingly popular in western and European countries (19), leading to the development of different schools of Yoga and an increase in the use of Yoga-based interventions in health research (20). Research evidence suggests that Yoga interventions help reduce stress, anxiety, depression, and musculoskeletal pain among HCPs and medical students at high risk of compromised health (23). Findings from a systematic review suggested that Yoga may be an effective approach for managing and preventing stress and burnout in HCPs (24).

While the effects are evident, Yoga-related studies have identified adherence to intervention as a limiting factor due to a lack of motivation, the effort required in practice, and time constraints (25), all three of which seem to be common phenomena among HCPs who work hectic schedules and continuous shifts. Thus, compliance becomes a major issue in behavioral interventions like Yoga (26).

mHealth, or mobile health, refers to the use of mobile devices such as smartphones, tablets, and wearable technology for health-related purposes (26). mHealth has been effective in improving care services, especially those aimed at changing behavior (27). mHealth has been effective in facilitating behavioral changes related to disease prevention, lifestyle modification, and disease management (28). The use of mHealth for self-monitoring and implementing lifestyle interventions has been shown to positively impact adherence to behavioral interventions (29, 30). Thus, mHealth can be a valuable technological tool for delivering behavioral interventions like yoga and addressing the problem of compliance.

Since physician health is a burning topic nowadays, especially after the COVID pandemic, global initiatives are being taken at healthcare institutions worldwide to implement evidence-based mind–body interventions for relieving job-related stress among doctors, nurses, and employees. India too is ramping up its use of these tried and tested indigenous tools of stress management in health research (31); however, methodological strength and robustness remain persistent issues (23, 24).

Considering the multi-modal effects of yoga-based interventions (32), this study was planned as a stepping stone toward addressing the hot topic of burnout among Indian HCPs. Accounting for the feasibility and ease of access provided by mHealth, it was decided to deliver the intervention online. This study examined changes in MBI and PQL outcomes among HCPs before and after a 12-week mHealth-aided yoga-based breath and meditation intervention in comparison to a waitlist control group.

## 2. Methodology

### 2.1. Study design and population

This was a randomized, waitlist-controlled trial with a 1:1 allocation ratio, utilizing a simple random sampling method for recruiting eligible participants. The study commenced after obtaining due approval from the institutional ethics committee (AIIMS/IEC/20/733) and registration with the Clinical Trial Registry of India (CTRI/2020/11/029136).

The population for this study consisted of HCPs, including faculty members, resident doctors, medical students, nursing officers, and nursing staff posted at high dependency units, critical care units, intensive care units, and emergency departments of a tertiary care hospital in north India.

### 2.2. Participant enrolment

Infographic posters containing study procedures and contact details of the investigators were attached to the notice boards of the designated areas and circulated within the social media groups shared by HCPs. Interested candidates were contacted by phone after duty hours and briefed in detail about the study procedures.

Adult HCPs (18–60 years of age) employed at the host institute for the past six months were included after confirming free accessibility of mobile or a laptop device with good internet connectivity. Pregnant females and individuals already practicing yoga, meditation or any other stress reduction practice were not included. Participants were also excluded if reported having medical conditions like musculoskeletal disorders, uncontrolled hypertension, diabetes, cardiovascular disease, epilepsy, bipolar disorder, or schizophrenia.

**Figure 1:**
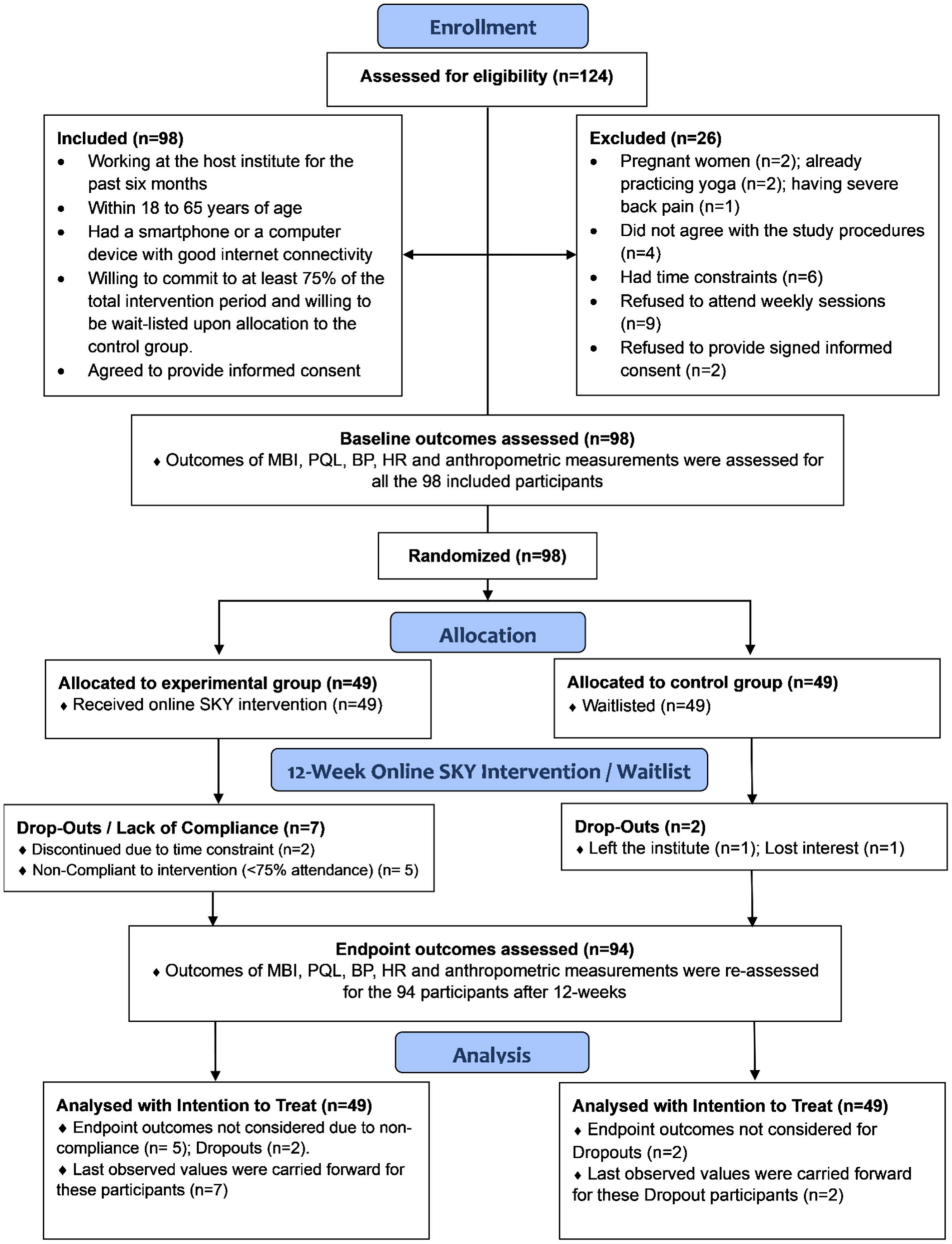
Consort flow diagram.

A total of 124 individuals who showed interest were assessed for eligibility, and 98 participants were enrolled sequentially from March 2021 to November 2022. Figure 1 depicts the flow of events during enrollment (33).

### 2.3. Randomization

Eligible participants were randomly allocated to the Experimental and Control groups in a 1:1 ratio using a computer-generated random sequence. Allocation concealment was implemented using sequentially numbered, opaque, sealed envelopes. Baseline outcomes were assessed after randomization.

### 2.4. Participants’ characteristics

**Figure 2:**
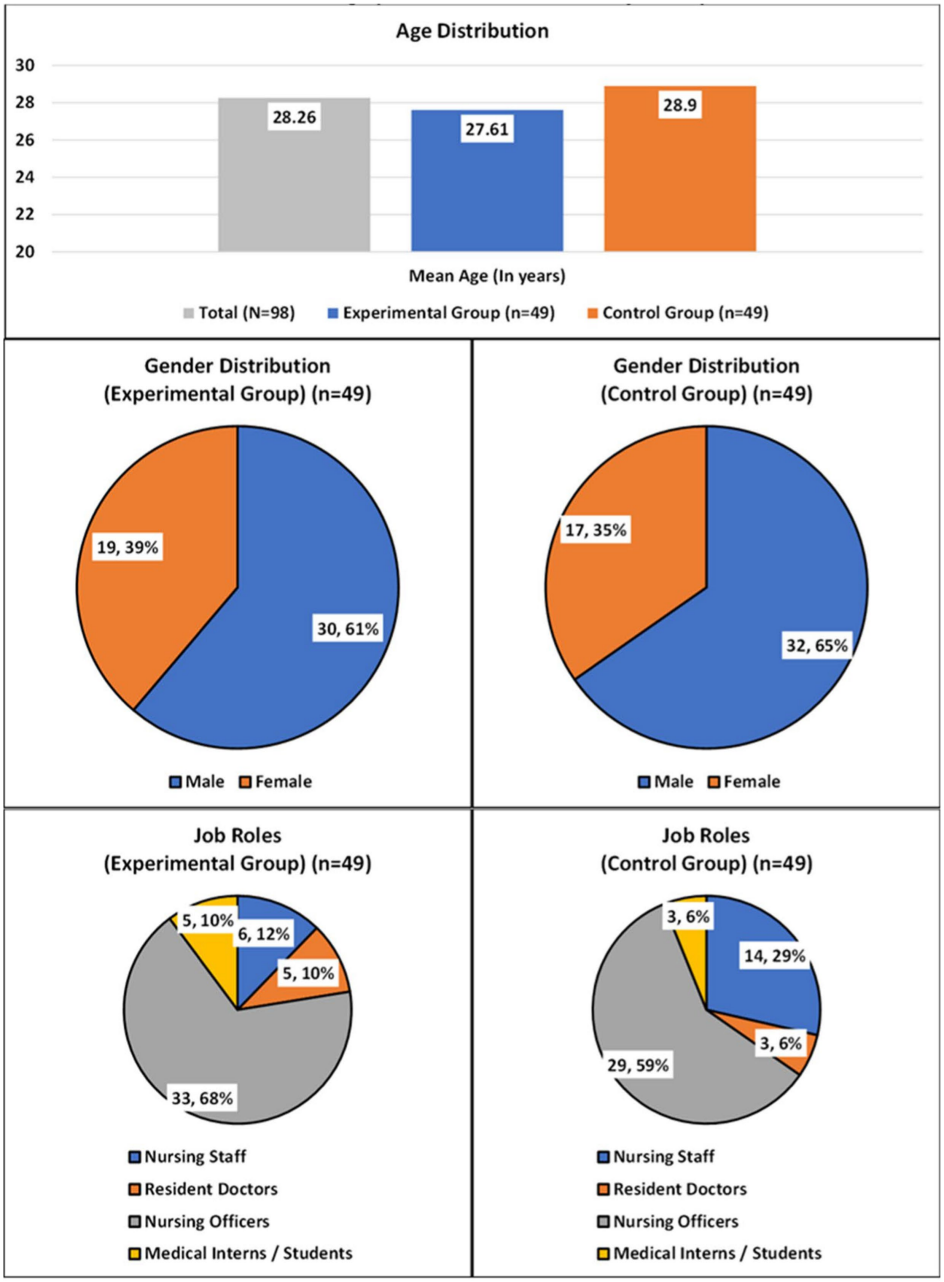
Demographic distribution across study population.

A total of 62 males and 36 females were enrolled. There were 30 males and 19 females in the experimental group, while the control group had 32 males and 17 females. The average age for all the participants was 28.26 ± 3.547 years (age range: 21 to 39 years), for the experimental group it was 27.61 ± 3.610 years, and for the control group it was 28.90 ± 3.399 years. Both groups were comparable at baseline in terms of demographic variables like gender, age and job-role. See Table 1 and Figure 2 for details on the demographic distribution across the study population.

**Table 1:** Demographic distribution across study population.

Demographic variable type and name		TOTAL (*N* = 98)	Experimental Group (*n* = 49)	Control (*n* = 49)	*p* between groups
**Gender** **(Counts & Percentage)**	**Female**	36 (36.73%)	19 (38.77%)	17 (34.69%)	0.417^*^
	**Male**	62 (63.26%)	30 (61.22%)	32 (65.30%)	
**Job Role** **(Counts & Percentage)**	**Resident Doctor**	8 (8.16%)	5 (10.20%)	3 (6.12%)	0.216^#^
	**Medical Student**	8 (8.16%)	5 (10.20%)	3 (6.12%)	
	**Nursing Officer**	62 (63.26%)	33 (67.34%)	29 (59.18%)	
	**Nursing Staff**	20 (20.40%)	6 (12.24%)	14 (28.57%)	
**Age in years (Mean & SD)**		28.26 ± 3.547	27.61 ± 3.610	28.90 ± 3.399	0.73^$^

*Based on Chi-Square Test; $Based on Independent T test; #Based on Fisher’s Exact Test.

### 2.5. Outcome measures

#### 2.5.1. Maslach burnout inventory

The Maslach Burnout Inventory (MBI) – Human Services Survey for Medical Personnel (HSS-MP) (34) was used to measure Burnout. The tool is intended specifically for HCPs and has 22 statements based on subjective feelings and viewpoints. The inventory is divided into three subscales: the Emotional Exhaustion (EE) subscale has nine items, the Depersonalization (DP) subscale has five, and the Personal Accomplishment (PA) subscale has eight. All 22 item statements are based on a seven-point Likert scale, with a frequency score of zero being never, one being a few times a year, two being once a month or less, three being a few times a month, four being once a week, five being a few times a week, and six being every day.

#### 2.5.2. Professional quality of life scale

The PQL scale was used for measuring levels of PQL (12). It has been extensively used since 1995 to assess the positive and negative aspects of working as a human service provider. It has 30 statements that are based on personal sentiments and attitudes and are equally divided into three sub-scales. The Compassion Satisfaction subscale (CS) rates the positive aspects of working as a helper; the Burnout (BO) subscale assesses work-related hopelessness and feelings of inefficacy; and the Secondary Trauma (ST) sub-scale evaluates secondary exposure to extremely or traumatically stressful workplace events. The PQL measures the frequency of each statement on a Likert scale as follows: one = never; two = rarely; three = sometimes; four = often; and five = very often.

#### 2.5.3. Secondary outcomes

Systolic Blood Pressure (SBP), Diastolic Blood Pressure (DBP), and resting Heart Rate (HR) were all measured using the Omron automatic blood pressure monitor HEM-7120 (35), with the participant seated comfortably in chair for five minutes prior to measurement. An average of three consecutive readings measured at intervals of five minutes was considered the final value of SBP, DBP, and HR. Height was measured using a stadiometer and weight using an electronic weighing machine. Body Mass Index (BMI) was calculated by dividing weight in kilograms by height in meters^2^ (using formula BMI = kg/m^2^). Waist Circumference (WC) and Hip Circumference (HC) were measured using a non-elastic measuring tape, and waist-to-hip ratio (WHR) was calculated by dividing WC by HC (using formula WHR = WC/HC).

All outcomes were assessed at baseline and after 12 weeks, i.e., at endpoint. Observations were recorded on a case report form, and assessments were carried out in a safe environment in line with the COVID protocol for disease prevention.

### 2.6. Intervention details

The mHealth-aided 12-week yoga-based meditation and breath Intervention was implemented using an online video conferencing application, “Zoom Meetings” (Version: 5.7.7 (1105) (zoom.us)). The intervention was based on Sudarshan Kriya, a comprehensive breathing technique that involves Ujjayi pranayama, Bhastrika pranayama, Om chanting, and rhythmic breathing, followed by resting in Shavasana. The entire 12-week intervention was divided into the following three main components:

#### 2.6.1. Four-day orientation

Highly trained instructors from the Art of Living organization led the initial four-day orientation program online. Each of the four sessions lasted two hours per day and included practical demonstrations to train participants. On the fourth day, participants received instructions for home practice.

#### 2.6.2. Home practice

Participants performed the practice at home once a day, every six days a week, which lasted approximately thirty minutes per session. Participants were assisted in their practice online during three designated time slots: morning, afternoon, and evening. Participants were free to choose from any of the three time slots according to their availability.

#### 2.6.3. Guided weekly online sessions

Participants attended weekly online group sessions, during which, the Yoga instructors and the investigators emphasized the importance of regular practice.

Regular practice reminders were sent to participants in the form of mobile text messages and phone calls. Participants’ attendance logs were maintained for both home practice and weekly sessions. A minimum of 75% compliance was necessary for participants to be eligible for the final evaluation.

### 2.7. Statistical methods

Continuous variables were described using means and standard deviations. Counts and percentages were used to describe categorical variables. Continuous variables were checked for normal distribution using the Shapiro–Wilk test of normality, and parametric and non-parametric statistical tests were applied accordingly. An independent t test was used between two groups, and a paired t test was used within the same group for normally distributed outcome variables. For non-normally distributed outcome variables, the Wilcoxon Rank Sum test, also known as the Mann–Whitney u test, was used between two groups, while the Wilcoxon Signed Rank test was used within the same group. The Chi-square test was used to assess compliance within groups. A *p* value of ≤0.05 was considered as statistically significant. Effect size was estimated for all outcomes based on Cohen’s *d* using G*Power (Ver. 3.1.9.4) (36) and was interpreted as small (≥0.20), medium (≥0.50), and large (≥0.80) magnitudes (37). Post-hoc analysis of power was performed to check for type 2 error in primary endpoint outcomes between groups using OpenEpi (Version 3). All data was stored in “Microsoft Excel” (Version 2019) (Microsoft Corporation). Statistical tests were performed using “IBM-SPSS Statistics” (Version 26) (IBM Corporation, Armonk, New York). An ‘intention to treat’ analysis with a ‘last observed value carried forward’ approach was followed to minimize attrition bias. A sample size of 74 participants was determined using G*Power (36) based on findings from a previous study (38). An attrition rate of 20% was added to this, raising the total sample size to be 90, with 45 participants in each group.

## 3. Results

### 3.1. Intergroup analysis (experimental group vs. control group)

A highly significant difference (*p* < 0.001) was observed after 12-weeks between the two groups for all the outcomes of MBI and PQL, depicting a large effect (Cohen’s *d* > 0.8) for EE, DP, PA, CS, BO, and ST (See Table 2).

**Table 2:** Intergroup comparison of outcomes of maslach burnout inventory and professional quality of life.

Outcome variable	Baseline				Endpoint			
	Experimental group (*n* = 49)	Control group (*n* = 49) Baseline	*p*	*d*	Experimental group (*n* = 49)	Control group (*n* = 49) Baseline	*p*	*d*
**MBI Emotional Exhaustion**	23.90 ± 9.982	25.49 ± 12.942	0.299^#^	0.137	17.65 ± 9.291	26.27 ± 10.204	0.000^*$^	0.883
**MBI Depersonalization**	8.96 ± 4.335	9.33 ± 5.505	0.549^#^	0.074	6.02 ± 4.049	10.18 ± 5.118	0.000^*#^	0.901
**MBI Personal Accomplishment**	37.14 ± 5.679	37.98 ± 5.725	0.469^$^	0.147	41.16 ± 5.076	36.78 ± 4.980	0.000^*#^	0.871
**PQL Compassion Satisfaction**	38.51 ± 5.276	37.57 ± 4.659	0.353^$^	0.188	42.67 ± 4.543	37.00 ± 4.036	0.000^*$^	1.319
**PQL Burnout**	24.80 ± 5.519	26.67 ± 6.678	0.081^#^	0.305	20.80 ± 4.945	27.02 ± 5.282	0.000^*#^	1.215
**PQL Secondary Trauma**	23.73 ± 5.167	25.31 ± 6.056	0.170^$^	0.280	20.02 ± 4.100	25.71 ± 5.012	0.000^*#^	1.242

*Significant value (< 0.05); $Based on Independent T test; #Based on Mann–Whitney U test; Cohen’s *d* describes the effect size of differences between groups.

MBI, Maslach Burnout Inventory; PQL, Professional Quality of Life.

Post-hoc analysis of primary endpoint outcomes depicted a power of 99.21, 99.38, 99.07% for EE, DP and PA, while a 100% for CS, BO and ST, respectively (See Table 3). No significant difference was observed after 12-weeks for the secondary outcomes of SBP (*p* 0.135), DBP (*p* 0.286), HR (*p* 0.117), Weight (*p* 0.207), Height (*p* 0.598), BMI (*p* 0.282), HC (*p* 0.137), and WHR (*p* 0.172) (See Table 4).

**Table 3:** Post-hoc analysis of power for endpoint outcomes of maslach burnout inventory and professional quality of life.

Outcome variable	Statistic	Experimental group	Control group	Mean difference*	Power
**MBI Emotional Exhaustion**	**Mean**	17.65	26.27	−8.62	99.21%
	**Sample size**	49	49		
	**Standard deviation**	9.291	10.204		
	**Variance**	86.3227	104.122		
**MBI Depersonalization**	**Mean**	6.02	10.18	−4.16	99.38%
	**Sample size**	49	49		
	**Standard deviation**	4.049	5.118		
	**Variance**	16.3944	26.1939		
**MBI Personal Accomplishment**	**Mean**	41.16	36.78	4.38	99.07%
	**Sample size**	49	49		
	**Standard deviation**	5.076	4.98		
	**Variance**	25.7658	24.8004		
**PQL Compassion Satisfaction**	**Mean**	42.67	37	5.67	100%
	**Sample size**	49	49		
	**Standard deviation**	4.543	4.036		
	**Variance**	20.6388	16.2893		
**PQL Burnout**	**Mean**	20.8	27.02	−6.22	100%
	**Sample size**	49	49		
	**Standard deviation**	4.945	5.282		
	**Variance**	24.453	27.8995		
**PQL Secondary Trauma**	**Mean**	20.02	25.71	−5.69	100%
	**Sample size**	49	49		
	**Standard deviation**	4.1	5.012		
	**Variance**	16.81	25.1201		

*Mean difference = (Experimental Group mean) - (Control Group mean).

MBI, Maslach Burnout Inventory; PQL, Professional Quality of Life.

**Table 4:** Intergroup comparison of secondary outcomes of blood pressure, heart rate and anthropometrics.

Outcome variable	Baseline				Endpoint			
	Experimental group (*n* = 49)	Control group (*n* = 49)	*p*	*d*	Experimental group (*n* = 49)	Control group (*n* = 49)	*p*	*d*
**Systolic BP**	119.88 ± 10.686	120.98 ± 9.780	0.596^$^	0.107	119.33 ± 8.125	122.02 ± 9.508	0.135^$^	0.412
**Diastolic BP**	79.69 ± 8.725	78.35 ± 8.092	0.430^$^	0.159	78.80 ± 6.529	80.33 ± 7.556	0.286^$^	0.216
**Heart Rate**	83.37 ± 8.951	80.39 ± 8.563	0.095^$^	0.340	78.57 ± 6.696	80.96 ± 8.190	0.117^$^	0.319
**Weight**	63.657 ± 9.4501	66.157 ± 11.355	0.239^$^	0.239	64.054 ± 8.203	66.581 ± 11.259	0.207^$^	0.256
**Height**	165.343 ± 7.175	166.192 ± 8.931	0.605^$^	0.104	165.347 ± 7.171	166.214 ± 8.942	0.598^$^	0.106
**BMI**	23.233 ± 2.800	23.840 ± 2.936	0.298^$^	0.211	23.403 ± 2.447	24.003 ± 3.010	0.282^$^	0.218
**Waist Circumference**	84.090 ± 8.625	88.288 ± 9.774	0.026^a*^	0.455	84.207 ± 8.158	88.395 ± 9.869	0.024^$*^	0.462
**Hip Circumference**	92.888 ± 7.933	95.282 ± 7.729	0.134^$^	0.305	92.976 ± 7.757	95.343 ± 7.885	0.137^$^	0.306
**Waist to Hip Ratio**	0.906 ± 0.070	0.927 ± 0.080	0.180^$^	0.279	0.907 ± 0.067	0.927 ± 0.080	0.171^$^	0.271

*Significant value (< 0.05); $Based on independent T test; Cohen’s *d* describes the effect size of differences between groups.

BP, Blood Pressure; BMI, Body Mass Index.

### 3.2. Intragroup analysis (baseline vs. endpoint)

A highly significant difference (*p* < 0.001) was observed within the Experimental group for the outcomes of MBI and PQL, depicting a moderate effect (Cohen’s *d* 0.5–0.79) for EE, DP, PA, BO, and ST and a large effect for CS (Cohen’s *d* > 0.8). Within the control group, there was no significant difference observed for the outcomes of EE (*p* 0.51); CS (*p* 0.228); BO (*p* 0.294); and ST (*p* 0.533); however, DP (*p* 0.001) increased significantly and PA (*p* 0.018) reduced significantly (see Table 5). A significant reduction was also observed in HR within the experimental group (*p* < 0.001) but not in the control group (*p* 0.124). SBP (*p* 0.001) and DBP (*p* < 0.001) increased significantly within the control group, but no significant change was observed within the experimental group (SBP: *p* 0.289; DBP: *p* 0.109) (See Table 6).

**Table 5:** Intragroup Comparison of Outcomes of Maslach Burnout Inventory and Professional Quality of Life.

Outcome variable	Experimental group (*n* = 49)				Control group (*n* = 49)			
	Baseline	Endpoint	*p*	*d*	Baseline	Endpoint	*p*	*d*
**MBI Emotional Exhaustion**	23.90 ± 9.982	17.65 ± 9.291	0.000^*#^	0.647	25.49 ± 12.942	26.27 ± 10.204	0.51^$^	0.066
**MBI Depersonalization**	8.96 ± 4.335	6.02 ± 4.049	0.000^*$^	0.700	9.33 ± 5.505	10.18 ± 5.118	0.001*^$^	0.159
**MBI Personal Accomplishment**	37.14 ± 5.679	41.16 ± 5.076	0.000^*$^	0.744	37.98 ± 5.725	36.78 ± 4.980	0.018*^#^	0.222
**PQL Compassion Satisfaction**	38.51 ± 5.276	42.67 ± 4.543	0.000^*#^	0.840	37.57 ± 4.659	37.00 ± 4.036	0.228^#^	0.130
**PQL Burnout**	24.80 ± 5.519	20.80 ± 4.945	0.000^*#^	0.761	26.67 ± 6.678	27.02 ± 5.282	0.294^$^	0.042
**PQL Secondary Trauma**	23.73 ± 5.167	20.02 ± 4.100	0.000^*$^	0.785	25.31 ± 6.056	25.71 ± 5.012	0.533^$^	0.071

*Significant value (< 0.05); ^$^Based on Wilcoxon Signed Ranks test; ^#^Based on Paired T test; Cohen’s *d* describes the effect size of differences within groups.

MBI: Maslach Burnout Inventory; PQL: Professional Quality of Life.

**Table 6:** Intragroup comparison of secondary outcomes of blood pressure, heart rate and anthropometrics.

Outcome variable	Experimental group (*n* = 49)				Control group (*n* = 49)			
	Baseline	Endpoint	*p*	*d*	Baseline	Endpoint	*p*	*d*
**Systolic BP**	119.88 ± 10.686	119.33 ± 8.125	0.289^$^	0.056	120.98 ± 9.780	122.02 ± 9.508	0.001^$^	0.107
**Diastolic BP**	79.69 ± 8.725	78.80 ± 6.529	0.109^$^	0.113	78.35 ± 8.092	80.33 ± 7.556	0.000^$^	0.247
**Heart Rate**	83.37 ± 8.951	78.57 ± 6.696	0.000^$^	0.595	80.39 ± 8.563	80.96 ± 8.190	0.124^$^	0.067
**Weight**	63.657 ± 9.4501	64.054 ± 8.203	0.215^$^	0.446	66.157 ± 11.355	66.581 ± 11.259	0.064^$^	0.037
**Height**	165.343 ± 7.175	165.347 ± 7.171	0.322^$^	0.000	166.192 ± 8.931	166.214 ± 8.942	0.237^$^	0.002
**BMI**	23.233 ± 2.800	23.403 ± 2.447	0.152^$^	0.064	23.840 ± 2.936	24.003 ± 3.010	0.054^$^	0.054
**Waist Circumference**	84.090 ± 8.625	84.207 ± 8.158	0.474^$^	0.013	88.288 ± 9.774	88.395 ± 9.869	0.224^$^	0.010
**Hip Circumference**	92.888 ± 7.933	92.976 ± 7.757	0.626^$^	0.011	95.282 ± 7.729	95.343 ± 7.885	0.418^$^	0.007
**Waist to Hip Ratio**	0.906 ± 0.070	0.907 ± 0.067	0.725^$^	0.014	0.927 ± 0.080	0.927 ± 0.080	0.446^$^	0.007

*Significant value (< 0.05); $Based on Paired T test; Cohen’s *d* describes the effect size of differences within groups.

BP: Blood Pressure; BMI: Body Mass Index.

## 4. Discussion

The results show that after mHealth aided 12-week yoga-based meditation and breath intervention, all MBI and PQL outcomes improved for the experimental group HCPs. Previous studies have investigated the efficacy of various yoga and mindfulness-based interventions on outcomes of burnout among HCPs (38–44). They showed significant improvements in either one or two aspects of burnout in the yoga, mindfulness, and meditation-related intervention groups when compared to their respective control groups (38, 40–43).

Two studies showed improvements in EE and DP but not in PA (38, 41). One study showed improvements in EE alone (44). One study showed improvement in DP but not in EE and PA (40). In another study, improvements were observed in EE and PA but not in DP (43). A triple arm study showed improvement only in EE for the yoga group, and in EE and PA for the meditation group (42). However, it used the Japanese Burnout Index, which is similar to MBI but adapted for the Japanese populations (42).

Only one study showed improvements in all three outcomes of MBI, i.e., EE, DP, and PA (39). However, the intervention used in this study was of a shorter duration, i.e., four weeks, and included Laughter Yoga, which is performed in a group (39).

Regarding PQL, one study showed improvements in CS and BO within the yoga intervention group but not when compared to the control group (40).

The following section provides a closer look at the individual outcomes, possible mechanisms behind yoga’s effect, and the implications of each of the outcomes for HCPs:

### 4.1. Emotional exhaustion

EE involves energy drain, resulting in a feeling of being stressed and emotionally overwhelmed (34). Our results suggest that 12 weeks of yoga-based breath and meditation intervention significantly reduced EE among HCPs. This is supported by the findings of other yoga-based intervention studies (38, 39, 41–44). This decrease in EE can be attributed to the increase in parasympathetic drive due to regular practice of yoga, which helps calming down the stress response systems (32).

Yoga breathing modulates the hypothalamic–pituitary axis *via* hypothalamic and anterior pituitary vagal afferents (45, 46). Yoga practice brings about a state of calm alertness (32). Yoga also helps increase the production of well-being hormones like prolactin, oxytocin, and vasopressin (45, 46). It decreases production of stress hormones like cortisol and adrenocorticotropin while optimizing serum brain-derived neurotrophic factor levels (45, 46). Therefore, regular yoga practice might play a significant role in alleviating EE.

HCPs with high levels of EE are more likely to develop physical and mental health issues, further compromising patient care (1). Eventually, if EE is not managed, it may hinder patient care and decision-making (34). Our results add to the evidence that meditation and breathing practices can reduce both work-related exhaustion (47) and EE (48).

### 4.2. Depersonalization

The present findings align with previous studies which have also reported a notable decrease in DP after participants underwent a yoga-based intervention. (38–41)

Yoga based practices have been shown to increase empathy (49–51), consciousness quotient (49), mindfulness (52–54), enhance prosocial behaviors (50, 55), and increase self-compassion (50, 54, 55). These benefits may also result in reducing DP.

HCPs with lower levels of DP and greater empathy can recognize and respond better to patient emotions and needs (56). On the contrary, when HCPs have high levels of DP, it can cause them to see patients as objects or tasks instead of individuals in need of care (1). This can lead to a decrease in patient-provider trust and rapport, ultimately compromising the delivery of healthcare (34).

Yoga based interventions have also been found to reduce interpersonal disengagement (47), cynicism (48), self-role distance (48), and improve social connectedness (57). These improvements can also be attributed to a reduction in DP. Whereas, high levels of DP can result in a lack of empathy, detachment from work, and work-related cynicism (34) and can add to lower job satisfaction, increased absenteeism, negligence, and even medical errors (1).

### 4.3. Personal accomplishment

PA involves a sense of making an impact on one’s work and the people around them (34). In the current study, PA increased significantly for the yoga based intervention group, which is in line with the past findings (39, 42, 43). Literature also suggests that meditation and breathing practices increase professional fulfillment (47), professional efficacy (48), and have been associated with an increase in life satisfaction and self-esteem (58).

Yoga based practices have been shown to increase overall brain wave activity, increase gray matter, and enhance amygdala and prefrontal cortex activation (59). This increases interhemispheric brainwave synchronization, improves memory, attention, emotional control, and cognitive state (60).

Furthermore, yoga practice positively affects autonomic tone (61–63), improves heart rate variability (64), and enhances sleep parameters (64, 65), all of which are crucial for work-related productivity. Thus, regular yoga practices can help HCPs be more productive in their workplace and help achieve a sense of accomplishment and fulfillment.

When HCPs feel satisfied, it improves the quality of care they provide (34). This in turn improves organizational culture, increases job satisfaction, and reduces employee turnover (1, 6). On the other hand, low PA might cause HCPs to lose motivation (1), further hampering productivity, job satisfaction, and diminish patient care (1).

### 4.4. Compassion satisfaction

CS is about the pleasure one derives from being able to do one’s work well (12). Our study adds to past findings showing improvements in CS (40). Literature also suggests that yoga-based interventions can enhance professional fulfillment through self-worth, satisfaction, and a sense of contribution (47).

High levels of CS can make HCPs feel happier and more fulfilled with their work (12). This results in job satisfaction, motivation, better engagement, productivity, and quality care (40). Whereas, low CS can leave HCPs feeling exhausted, unfulfilled, and disconnected from their positive work (13). It adversely affects job satisfaction, care, and motivation (13). Eventually, HCPs may lose purpose and meaning in their work, decreasing their commitment and increasing turnover (1).

### 4.5. Burnout

BO is one of the two prime components of compassion fatigue (12). BO is generally associated with a high workload and a non-supportive environment (40). The present results are in line with previous findings suggesting that yoga-based interventions can help improve BO (40, 57) Yoga also improves parameters of sleep, life satisfaction, and resilience (65), all three of which are inversely related to Burnout.

Yoga improves emotional awareness, which increases resilience by enhancing self-awareness and empathy for others (66). Yoga-based practices help increase interoception (67) and develop emotional self-regulation and awareness. Their regular practice improves selective intentional attention and helps develop a non-judgmental attitude toward one’s own experiences (65). This insight helps combat negative self-perceptions and help reduce self-criticism, negative reappraisal, emotional reactivity, and rumination (65) which play a crucial role in overcoming BO.

Since BO causes a gradual onset of hopelessness and a decline in job performance, it gets reflected by self-criticism and a feeling of lack of efficacy (12). Thus, yoga-based interventions might help HCPs to be self-aware and avoid self criticism. Simultaneously, it can help enhance their psychological resilience and enable them to better manage workload, even in challenging circumstances like viral outbreaks (65, 68).

### 4.6. Secondary trauma

ST refers to the second component of compassion fatigue and involves secondary exposure to traumatic experiences at work (12). Our results of a significant reduction in ST for the experimental group are novel when compared to past studies (40).

Evidence suggests Meditation and yoga breathing practices as effective tools in reducing anxiety and post-traumatic stress (68–70). Patients with post-traumatic stress disorder (PTSD) benefit from yoga-based practices in terms of emotional and physical resilience, stress, and energy (71–78). Yoga helps reduce PTSD symptoms by reducing stress-induced allostatic load and increases parasympathetic activity, leading to a decrease in physiological arousal (71).

Additionally, yoga reduces physical tension, increasing relaxation, attenuating automatic thoughts and behaviors associated with hyperarousal (72), thus enabling practitioners to achieve a state of calm and restfulness (55).

HCPs tend to face ST on a more regular basis in comparison to other occupational professionals (40). If unhandled, ST may cause fear, intrusive images and sleep problems (1). ST can impair an HCP’s well-being, increase their risk of mental health disorders, and reduce their ability to provide empathetic and effective care (1).

Considering yoga’s beneficial effects on psychological trauma, HCPs can benefit from yoga-based practices to overcome the negative effects of working as a caregiver, thereby preventing compassion fatigue and protecting one’s mental health.

### 4.7. Other findings

Our study showed a significant reduction in HR within the meditation and yoga breathing group. This is a common finding for yoga based interventional studies (32, 45, 46, 64). Since all the participants were healthy adults, anthropometrics did not vary significantly post-intervention. However, a significant change in DP, PA, SBP, and DBP was observed within the waitlist-control group participants. These could be due to the stressful and demanding nature of participants’ jobs (1, 4, 5, 9–11, 79), as DP and PA are both components of Burnout, while stress is identified as a significant risk factor for increase in BP.

### 4.8. Strengths, limitations, and future directions

Yoga-based research studies suffer from shorter interventions and low compliance rates. This impacts the treatment effect of yoga, ultimately diminishing the overall evidence on yoga’s efficacy. However, our study used a longer intervention of 12-weeks, in comparison to past studies which ranged from four to eight weeks of intervention (38–44).

Longer interventions are associated with greater improvements in outcomes of psychological well-being and behavioral functions (80). This might be the reason for a greater effect on outcomes of our study, when compared to past research (38–44).

The mHealth-aided intervention allowed HCPs to choose their preferred time and space for practice, enabling them to incorporate it into their busy schedules. Further research can help establish the treatment fidelity of mHealth-aided yoga-based interventions.

Our study utilized a unique and thoroughly-researched yoga-based technique called Sudarshan Kriya (32, 45–47, 61–63, 70, 75). It follows a specific sequence of procedures that can be taught by trained individuals and implemented at home using simple and easy to follow instructions, therefore, making it suitable for prescription purposes. Since this was an online based intervention, the long-term effects of offline or in-person Sudarshan kriya practice also need to be explored in the context of Burnout and PQL.

Our study lacked blinding among the two groups and an active intervention for the comparator group. Future research can be strengthened by implementing blinding of participants and employing an active or sham intervention for the control group.

Researchers are also encouraged to utilize objective psychological, physiological, neurological, endocrine, and immunological outcome measures for deriving possible correlations with the domains of MBI and PQL. Further research is warranted to generalize our findings.

## 5. Conclusion

The mHealth aided intervention showed a strong effect during intergroup comparisons and a moderate effect during within-group comparisons. The present evidence supports the use of Sudarshan Kriya Yoga for managing stress and building resilience among healthcare providers. It yielded significant improvements in outcomes of burnout and professional quality of life after 12 weeks. Current findings underscore the significance of integrating accessible and technology-enhanced yoga-based practices into the daily routines of healthcare professionals. Such integration can offer promising avenues toward a more sustainable and fulfilling healthcare ecosystem for both providers and patients alike. Further research in diverse healthcare settings and populations will undoubtedly refine and expand upon these promising outcomes, propelling us toward a brighter and more resilient future for healthcare providers worldwide.

## Data availability statement

The raw data supporting the conclusions of this article will be made available by the authors, without undue reservation.

## Ethics statement

The studies involving humans were approved by All India Institute of Medical Sciences (AIIMS) Rishikesh – Institutional Ethics Committee (IEC). The studies were conducted in accordance with the local legislation and institutional requirements. The participants provided their written informed consent to participate in this study. Written informed consent was obtained from the individual(s) for the publication of any potentially identifiable images or data included in this article.

## Author contributions

PB: Conceptualization, Data curation, Formal analysis, Investigation, Methodology, Project administration, Software, Visualization, Writing – original draft. MP: Conceptualization, Data curation, Funding acquisition, Investigation, Methodology, Project administration, Supervision, Writing – original draft. YB: Conceptualization, Data curation, Formal analysis, Funding acquisition, Software, Writing – original draft. DK: Project administration, Resources, Writing – review & editing. PH: Project administration, Resources, Writing – review & editing. VR: Writing – review & editing, Visualization.

## Funding

The author(s) declare financial support was received for the research, authorship, and/or publication of this article. This study was funded by the Government of India’s Department of Science and Technology’s (DST) scheme: “Science and Technology of Yoga and Meditation” (SATYAM).

## Conflict of interest

The authors declare that the research was conducted in the absence of any commercial or financial relationships that could be construed as a potential conflict of interest.

## Publisher’s note

All claims expressed in this article are solely those of the authors and do not necessarily represent those of their affiliated organizations, or those of the publisher, the editors and the reviewers. Any product that may be evaluated in this article, or claim that may be made by its manufacturer, is not guaranteed or endorsed by the publisher.
